# Evaluation of health care providers’ use of the ‘Exercise and Depression Toolkit’: a case study

**DOI:** 10.1186/s12888-021-03248-5

**Published:** 2021-05-08

**Authors:** Krista Glowacki, Daniah Zumrawi, Erin Michalak, Guy Faulkner

**Affiliations:** 1 Department of Kinesiology, Faculty of Education, University of British Columbia, 210-6081 University Boulevard, Vancouver, BC V6T 1Z1 Canada; 2 Department of Psychiatry, Mood Disorders Centre, Faculty of Medicine, University of British Columbia, 420-5950 University Boulevard, Vancouver, BC V6T 1Z3 Canada

**Keywords:** Exercise, Depression, Health care providers, Acceptability, Perceived effectiveness, Evaluation, Implementation, Behaviour change

## Abstract

**Background:**

Exercise is now recommended as a primary treatment for mild-moderate depression in Canada. The ‘Exercise and Depression Toolkit’ was developed to help health care providers (HCP) integrate these treatment guidelines into practice. The purpose of this study was to evaluate acceptability and perceived effectiveness of the toolkit in practice by HCPs working with individuals with depression.

**Methods:**

A case study design was utilized. The toolkit was given to 6 HCPs to use in practice for 4 weeks. Pre- and post-intervention phone interviews were conducted, and weekly logs were provided to track use and satisfaction of interactions with individuals with depression when using the toolkit. The study was conceptually guided by a hybrid theoretical approach using the Diffusion of Innovation Theory and the Theoretical Framework of Acceptability.

**Results:**

All HCPs used the toolkit at least once. Participants viewed their interactions when using the toolkit to be successful (considering individuals’ receptiveness, its usefulness and general satisfaction.) The average success score for all participants was 5.5/7. HCPs found the toolkit to be acceptable. All participants (*n* = 6) viewed the toolkit as having relative advantage in helping them to discuss exercise with individuals with depression, and as relatively simple and easy to use (not complex) and adaptable to their practice needs (having trialability). Participants liked the toolkit and had mostly positive things to say about it. Participants had mixed feelings about whether changes in the people they worked with (such as mood and activity levels) could be observed (observability) and whether the toolkit changed their belief in their ability to recommend or discuss exercise (self-efficacy). Recommended dissemination strategies were adopted in promoting the toolkit.

**Conclusions:**

Future work should address observability and the ability for health care providers to see other providers using it, as well as effectiveness considering outcomes for people with depression such as mood and activity changes. The results of this initial evaluation seem promising for uptake and future adoption of the toolkit by health care providers working with adults with depression in Canada.

**Supplementary Information:**

The online version contains supplementary material available at 10.1186/s12888-021-03248-5.

## Background

The Canadian Network for Mood and Anxiety Treatment (CANMAT) revised treatment guidelines in 2016 for Major Depressive Disorder (MDD) [1]. These guidelines recommend exercise as a first-line treatment for mild-moderate MDD, and as an adjunct treatment for moderate-severe MDD. In Canada, little structure exists for mental health professionals to explore exercise as a treatment option for adults with depression. Releasing guidelines or providing education about guidelines is not sufficient to elicit behaviour change [2]. Guidelines help to understand ‘what’ to do, but not ‘how’ to do it (implementation). Thus, the ‘Exercise and Depression Toolkit’ was developed using a theoretically informed and systematic process described in detail elsewhere [3] (publicly available at www.exerciseanddepression.ca). In summary, the toolkit was designed to address the barriers and facilitators to health care provider promotion of exercise [4] and for individuals with depression to participate in physical activity [5]. The primary purpose of the toolkit is to enable health care providers (HCP) to discuss and consider exercise as a treatment collaboratively with individuals with depression [3–6]. Now that the toolkit has been disseminated, monitoring knowledge use when translating health care knowledge to action is an important next step in the knowledge to action cycle [7]. In particular, examining how the toolkit is used in practice and whether it is considered acceptable by practitioners is necessary.

The Diffusion of Innovation (DoI) Theory helps explain the process by which people or groups adopt or reject an innovation which is a new idea, behaviour, or object [8]. The innovation itself, as well as its perceived attributes, may impact adoption and use of an innovation. The five attributes of an innovation considered important within this theory are relative advantage, compatibility, complexity, trialability and observability. See Table 1 for definitions of these attributes. If an adopter feels there is no relative advantage in using the innovation, it is not compatible with their values and beliefs or does not meet their needs, has a low degree of trialability and observability, or it is perceived as too complex, then it will not be adopted [8, 9]. These attributes were considered when developing the toolkit. Studies have also been done using the DoI theory to help understand adoption of interventions created for HCPs and health service delivery [9–12]. Overall, studies using Rogers’ DoI theory have identified that attributes of the innovation are important in the adoption of specific innovations in health care.

**Table 1:** Diffusion of Innovation definitions of attributes of an innovation

DoI Attribute	Definition
Relative Advantage	The degree to which innovation is perceived as better than a previous approach
Compatibility	The degree to which an innovation exists with values, past experiences and needs of potential adopters
Complexity	The degree in which an innovation is perceived as difficult to understand and use
Trialability	The degree to which an innovation may be experimented with or trialled on a limited basis
Observability	The degree to which the effects or results of an innovation are visible or able to be observed by the adopter

More recently, the Theoretical Framework of Acceptability (TFA) was developed to help assess the acceptability of health care interventions [13]. Acceptability can be defined as a “multi-faceted construct that reflects the extent to which people delivering or receiving a health care intervention consider it to be appropriate, based on anticipated or experienced cognitive and emotional responses to the intervention” (pg. 1) [13]. The TFA incorporates seven constructs of acceptability defined in Table 2. Acceptability of an intervention will impact successful implementation. If acceptability is considered low, the intervention may not be delivered as intended, which could impact other factors such as overall effectiveness [13, 14]. Qualitative studies have applied the TFA to assess intervention acceptability [15–17] and all deemed the theory to be helpful for understanding intervention implementation, identifying issues with a program, and informing changes in design to increase uptake.

**Table 2:** Definitions of the component constructs in the Theoretical Framework of Acceptability

TFA Construct	Definition
Ethicality	The extent to which the intervention has good fit with the individual’s value system.
Affective Attitude	How an individual feels about the intervention
Burden	The amount of effort required to participate in the intervention
Opportunity Costs	The benefits, profits and values that were given up to engage in the intervention
Perceived Effectiveness	The extent to which the intervention is perceived to have achieved or to achieve its intended purpose
Self-Efficacy	The participant’s confidence that they can perform the behaviour required to participate in the intervention
Intervention Coherence	The extent to which the participant understands the intervention and how it works

An innovation adoption process is not straightforward. Acceptability of the intervention to both intervention deliverers and intervention recipients will impact successful implementation [13, 14]. Understanding factors that could influence the adoption of new interventions is an important step for uptake and dissemination of an innovation. DoI theory is helpful for assessing perceived attributes of an intervention. The TFA compliments this theory by providing an in-depth conceptualization of acceptability that considers emotional and cognitive responses to an intervention that are not explicitly addressed by DoI theory. This hybrid complementary approach provides a new framework for evaluating the ‘Exercise and Depression Toolkit’.

The primary purpose of this study was to evaluate acceptability and perceived effectiveness of the toolkit in practice by HCPs working with individuals with depression. The secondary purpose was to attain feedback on the toolkit to determine necessary modifications and help inform national dissemination and uptake. Using a case study design [18], participants completed interviews before and after having access to the toolkit for 4 weeks. Use of the toolkit was logged over the four-week period.

## Methods

### Ethics and recruitment

Ethical approval was obtained from the University of British Columbia Ethics Board. Participants were recruited from across Canada through purposive sampling. HCPs that had participated in interviews during a previous phase of toolkit development were contacted and invited to participate in the study. This was done considering the DoI theory, as these individuals were considered likely to be innovators [8] of adopting the toolkit (the first to use it) as they have an identified interest in physical activity and mental health, and would be receptive to implementing the toolkit in their practice. Recruitment emails were also shared through private practice and public health authority networks.

### Eligibility

To be eligible for participation, HCPs had to be currently working with adults with depression, working a minimum of 2 days per week, and not have any planned vacation (for more than a week) during the four-week evaluation period. The toolkit was created for a variety of end users: HCPs who work with adults with depression such as Family Physicians, Psychiatrists, Counsellors, Mental Health Workers, Occupational Therapists, Recreation Therapists, Nurses, and Social workers. The aim was to recruit a sample inclusive of different end users from diverse professional designations.

### Data Collection

Case studies are commonly used to understand and explore professional practice behaviours [18] and in particular nursing practice in Canada [19]. A case study can be defined as an intensive, systematic investigation of a single individual, group, or community in which the researcher examines in-depth data [18]. Case studies are used to explore complex phenomena in a natural setting and are recommended as a research design to address how and why questions, and situations where a researcher has little control over manipulation of a behaviour [18]. The case study design allowed consideration of the individual health care providers and their context (e.g. family physician in primary care), as well as the providers as a group of ‘mental health professionals’. Within this design, the authors opted for multiple data collection methods to better understand health care providers’ practice behaviour in-depth.

Semi-structured telephone interviews were conducted by the first author (KG) pre- and post-intervention (after use of the toolkit for 4 weeks) scheduled at times convenient for participants. Participants were also given an electronic weekly log (that could be filled out by hand or online). The log was used to track use of the toolkit, as well as satisfaction with respect to their interactions with individuals with depression, and details of the ‘end point’ of what occurred after using the toolkit. Data collection occurred July 2019–October 2019 with rolling admission into the study. Interview guides and the weekly log are provided for reference as supplementary materials.

A pre-intervention interview guide was developed to help understand HCP’s practice before using the toolkit. During the pre-interview, a brief overview of how to use the toolkit was provided. Participants were then given four weekly logs to use at their discretion in their practice settings for 4 weeks. The log provided participants with an option to check and track the days that the toolkit was used and if a referral to an exercise program was made. Participants were asked to rate their perceived success of interactions with the individuals they work with when using the toolkit on a scale of 1–7 (1 being extremely low success, and 7 being extremely high success). Prompts were provided to consider success including usefulness, client receptiveness, and satisfaction. In-depth phone interviews were then conducted with all participants at the end of 4 weeks once all weekly logs were received. Questions addressed acceptability and prompts were included for each of the constructs of acceptability and the perceived attributes of an innovation. For example, for the acceptability construct ‘affective attitude’, participants were asked: what did you like about the toolkit, and what did you dislike about the toolkit? Final questions concerned the intent to continue using the toolkit, recommending it to colleagues, and possible dissemination strategies. Data collected from pre-intervention phone interviews and the weekly logs were used as prompts for these final interviews. Pre and post-intervention interview guides, as well as the weekly logs are available for reference as supplementary materials.

### Data Analysis

All interviews were audio-recorded and transcribed verbatim. Weekly logs were transferred or transcribed into one document for each participant. Logs, interviews, and transcriptions were assigned participant ID numbers to maintain confidentiality. Names were removed, and participants are referred to as their provider designation. Interviews ranged from 30 to 60 min in length. A content analysis was conducted to prepare, organize, and report on the data [20, 21]. A codebook was first created using the seven component constructs in the TFA [13] as well as the innovation attributes of the DoI [8] and their respective definitions. The concepts of adoption and dissemination were also included. Although coding was primarily deductive, there was flexibility in creating new codes if necessary. QSR International’s NVivo 11 Software was used to manage data and support qualitative data analysis. Coding was conducted collaboratively by the first and second author (KG and DZ) on one transcript and one weekly log to ensure understanding of the codes and concept definitions. After this collaborative approach, KG and DZ coded all transcripts independently and then met to review and discuss the coding framework. Any discrepancies in coding were discussed until agreement was reached, and when needed, the last author GF was consulted. Units of data were coded as statements which ranged from a sentence to a paragraph. Statements were considered new ideas or concepts, hence the variability in length of what was coded. For theoretical constructs, the authors coded statements as negative or positive. This was determined based on the participants’ description of views, context, and tone. A negative statement was considered something that would negatively impact adoption, and a positive statement would positively influence adoption. For example, if a participant felt that the toolkit was difficult and hard to understand and use, this was coded under complexity as a negative statement. The authors attempted to use one code to isolate the meaning of the text, versus double-coding passages of text. For example, participants were asked how they felt about the toolkit (their likes and dislikes). An example of a response commonly heard was “I just liked the simplicity, so, not too many pages, because that gets overwhelming for patients”. Such a response, stating it was simple or easy to use, was coded to complexity (as a positive statement) rather than complexity and affective attitude (how an individual feels about an intervention).

Various criteria were adopted to ensure ‘trustworthiness’ of this qualitative approach [21]. A critical reference group was consulted prior to conducting the study for data collection methods, evaluation of interview guide questions, and purposive sampling strategy [20–22]. Throughout the analysis process, two independent coders (KG & DZ) utilized a detailed pre-determined codebook and discussed meaning units and categorization throughout the analysis process [21]. Based on recommendations [21] the authors attempted to describe the analysis process in detail, with results reported systematically, carefully and with transparency as to how connections were made between the data and results.

## Results

Six HCPs participated in the study (see Table 3 for demographic information). Based on the data collected from weekly logs, all the HCPs (*n* = 6) used the toolkit at least once. Half (*n* = 3) of the participants referred individuals to an exercise program at least once. The number of times the toolkit was used (either part of it or the full toolkit) by each participant during the four-week evaluation period ranged from 1 to 40 times (median = 7.5; IQR = 5). The Occupational Therapist used it once and identified that she had recently taken on a managerial role splitting half of her working hours overseeing other practicing therapists which reduced her caseload and in turn reduced her opportunity to use the toolkit. The Psychotherapist used it 40 times. She explained that she had a low-cost exercise facility right next door to where she worked which reduced many barriers for her to use the toolkit and recommend exercise, thus enabling her to use it with almost all the people she saw with depression. Overall, participants perceived their interactions when using the toolkit with individuals with depression as successful. The average success score (range 1–7) of each participant of all their interactions ranged from 4.1–6.5. The average success score for all participants was 5.5/7. Table 4 provides a summary of results from the weekly logs.

**Table 3:** Demographic Characteristics

Gender, % (n)	
Female	100 (6)
Age in years	
M (SD)	43 (11)
Range	30–55
Education, % (n)	
Graduate School	100 (6)
Ethnicity, (n)	
Caucasian	5
Chinese	1
Employment Status, (n)	
Full-time	2
Part-time	4
HCP designation, (n)	
Family Physician	1
Nurse Clinician	1
Nurse Practitioner	1
Occupational Therapist	1
Psychotherapist	1
Social worker	1
Area of Professional Practice, (n)	
Outpatient Perinatal Mental Health	1
Primary care	1
Outpatient	2
Addiction Medicine	1
Community Private Practice	1
City Practicing In, (n)^a^	
Burnaby, BC	1
Squamish, BC	1
Sudbury, ON	1
Surrey, BC	1
Timmins, ON	1
Vancouver, BC	3
Experience w/ Adults w/ Depression in years	
M (SD)	14.2 (10.9)
Range	5–34
Diagnose Adults w/ Depression, % (n)	
No	83 (5)
Adults Seen w/ Depression Frequency, (n)	
Daily	1
Weekly	5
150 min PA/Week Completed, % (n)	
Yes	100 (6)

^a^Participants could identify more than one city of practice

**Table 4:** Summary of use of Toolkit in practice

Participant	# times full Toolkit used	# times part of Toolkit used	Total # times Toolkit used	Perceived success (average 1–7)	# of referrals to an exercise program
Nurse Clinician	1	7	8	5.8	1
Family Physician	4	0	4	5.5	0
Social Worker	0	9	9	5	0
Nurse Practitioner	5	2	7	4.1	0
Occupational Therapist	1	0	1	6	1
Psychotherapist	10	30	40	6.5	21

The results from coding of the post-interviews and weekly logs are presented in Table 5 for the theoretical constructs of the TFA [13] and DoI Theory [8]. Coded statements are presented as positive or negative statements to help understand adoption. Overall, all participants (*n* = 6) viewed the toolkit as having relative advantage and helping them to discuss exercise with individuals with depression. All participants viewed the toolkit as relatively simple and easy to use (not complex) and adaptable to their practice needs (having trialability). Participants liked the toolkit. With regards to observability, all participants identified one positive change they could see as a result of using the toolkit. However, half (*n* = 3) of the participants identified that they were not able to see any changes in some people (either they did not show up to follow-ups or did not follow through with goals). Half (*n* = 3) of participants identified that the toolkit did require some time and effort to use in practice (burden). With regards to self-efficacy, half of the participants (*n* = 3) identified that either the toolkit did not change their confidence to discuss exercise or that they did not feel as confident to use it with individuals who were not receptive to the consideration of exercise. A more detailed description of findings from the post-interviews and weekly logs as well as quotes that highlight results are provided below.

**Table 5:** Coding frequency in the component constructs of the TFA and DoI Theory from health care provider documents (interviews and weekly logs)

Theoretical Construct (DoI^**1**^ and TFA^**2**^)	Code Frequency positive (negative)	Number of documents with code positive (negative)	Number of participants with code positive (negative)
Relative Advantage^1^ and Perceived Effectiveness^2^	18 (1)	6 (1)	6 (1)
Compatibility^1^ and Ethicality^2^	13 (0)	7 (0)	6 (0)
Complexity^1^	19 (2)	7 (1)	6 (1)
Trialability^1^	18 (1)	8 (1)	6 (1)
Observability^1^	23 (4)	8 (3)	6 (3)
Affective Attitude^2^	37 (3)	7 (2)	6 (2)
Burden^2^	4 (3)	3 (3)	3 (3)
Opportunity Costs^2^	5 (1)	5 (1)	5 (1)
Self-efficacy^2^	9 (5)	5 (3)	5 (3)
Intervention Coherence^2^	5 (4)	4 (1)	4 (1)

^1^Theoretical construct from Diffusion of Innovation Theory

^2^Theoretical construct from Theoretical Framework of Acceptability

### Relative advantage and perceived effectiveness

All participants (*n* = 6) viewed the toolkit as helping them to discuss exercise more effectively than previous approaches. Participants explained that the toolkit reminded them to discuss exercise, that it was something tangible that could be given and used, and the evidence behind the recommendation was clear and well laid out. The Social Worker explained, “… if I didn’t have it, I wouldn’t ask. So, if the goal of the toolkit was to get me to engage with the women and talk about the relationship between exercise and depression, bring it up, have a conversation about it … if I didn’t have the toolkit, I would never have that conversation.”

### Compatibility and ethicality

All participants (*n* = 6) self-reported personally meeting the Canadian Physical Activity Guidelines. All participants viewed the toolkit and recommending exercise as aligning with their personal beliefs and values, as well as their beliefs and values as HCPs. Some participants also explained that the design (format and layout) and some general content of the toolkit aligned with how they were already practicing so this made it easy to transition into using it regularly. The Family Physician explained:

“The toolkit goes into my style of like how I like to run my office anyways, like I use a lot of drawn diagrams. I do like to make sure people understand, and I usually write down things for them. So, it wasn’t like, for me that whole kind-of process is not like – I prefer to practice like that, so I think – that’s why I found like, I had good, good experience with it.”

### Complexity

All participants stated that the toolkit was easy to use, understand and not complex. Some participants also identified that individuals with depression they were working with found it easy to use and understand as well, which reinforced the HCP’s positive evaluation of the toolkit and desire to continue using it. The Occupational Therapist explained:

“I liked that it was very simplistic. It’s not complex, it’s not too long, so, when you have a client, or have the client review the information, it’s not too challenging … like it was simple to use.”

Another participant identified that when she first received the toolkit, she had to concentrate when using it and that it was harder for her to make the connection between exercise and depression. She went on to explain in her post-interview that with time and continued use of the toolkit, it became easier.

### Trialability

Whether HCPs viewed the toolkit as being adaptable to their practice context was considered for trialability. All participants identified that different toolkit sections could be used as desired based on their context. The Nurse Practitioner included an unprompted comment in her weekly log after using the toolkit, “Individual previously had finances required for gym membership – this no longer the case. We discussed alternative plan which can be done @ home @ 0 cost: i.e. push-ups, crunches, squats, lunges, jump rope, biking, running, fast-paces walking”. This demonstrated her ability to use the toolkit with an individual in her practice, despite the fact that the individual she worked with had financial barriers and she was not able to refer to a structured exercise program or facility.

### Observability

Given the nature of the case study approach, it was not possible for participants to observe their peers use the toolkit in practice. In this context, we defined observability as ‘the extent to which the results of an evidence-based program become visible’ [23]. Participants identified that some of the individuals with depression reported they went and tried to exercise whether it was at home or in a structured class. From this, HCPs considered patients receptive to the idea of exercise, and some identified changes in mood and conversations on follow-up visits. The conversation with the Nurse highlighted this:

“I had another patient who wasn’t on the log, who brought me back some of the stuff filled in, yesterday actually.. . very basic, like she could only manage once a week walking to go get her kids from school. But that was better than zero, you know?”

Although the majority of statements within this construct were coded as positive, reasons for not observing any changes included lack of follow-up visits with people, individuals’ severity of depression with symptoms such as being unmotivated and tired.

### Affective attitude

For this construct, HCPs were asked specifically about their likes and dislikes. An overwhelming number of positive statements about the toolkit were expressed with and without prompting questions throughout the interviews and the logs. The Family Physician expressed:

“… the toolkit and like exercise and all that, is a good way to start that kind of – tap into all like the needs of the patient, as well as like, now with like evidence that can actually provide benefits … I think that was interesting. Like there’s something that you can like show them, you know, that it’s going to … like it’s proven to help, you know? Instead of just saying, ‘you exercise, you’ll feel better, you know?”

The Psychotherapist also expressed her positive feelings:

“But it was nice to actually have a handout on it, and actual tools that I can actually give to them, rather than say, ‘you know, exercise is great for depression’, it’s actually to have that toolkit at hand … and I think it was very beneficial for the clients too, because then they have something to take home.”

Three negative statements from two participants were about parts of the toolkit they personally did not use or find helpful rather than something they specifically disliked about the toolkit overall.

### Burden

Views of the HCPs were divided on how much time and effort was required to use the toolkit in practice. Participants were asked if they felt the toolkit required a lot of time and effort to use. Three participants viewed the toolkit as requiring little time and effort, as the Social Worker explained:

“No, because it’s just almost like bullet points. So, then when I’m talking to someone, I can say, ‘how does exercise help’, and then, there’s another sentence that I can just follow after that, you know. And when I’m talking to someone, having just those there very short bullet points, makes it a bit easier.”

Conversely, two participants felt that it does require time and effort, although this was minimal. The Occupational Therapist explained her views on the toolkit requiring more time and effort when working with new individuals:

“… where she was as a client, who was brand new, it might take a little – it would probably take a little bit more time in terms of being like, ‘okay, well, you know, this is how you’re feeling, these are the potential benefits of exercise, this is why it can add to your life’.”

### Opportunity costs

For opportunity costs, HCPs were asked if they felt that using the toolkit took away from other priorities they had (what they give up to use the toolkit in practice). Almost all (*n* = 5) participants felt that the toolkit did not take away from other priorities, and some explained that they felt exercise was a priority that they should be discussing. The Psychotherapist explained her priority of exercise: “Well, I always thought exercise is a priority of treating depression. So, I felt it enhanced that, because I would just have that conversation, and set some, you know, realistic goals with them”. On the other hand, the Nurse Practitioner said she felt that it did take away from other priorities:

“yes, it’s a little bit reprioritizing some of the workload … the time you take – yes. But just even the fact of, you know, pulling out the sheet, that’s – even if it’s embedded in your system, just going through that process of learning that, clicking on the right places, all that does cause some, you know, barriers. You know, we don’t like to change our ways”.

### Self-efficacy

HCPs were asked about their confidence in using the toolkit to discuss and recommend exercise, as well as if the toolkit had changed their confidence levels in general to discuss and recommend exercise with individuals with depression. The Family Physician explained that the toolkit helped her gain confidence in discussing exercise by providing conversational pieces:

“Yes, it kind of gives me like the … ‘trigger words’, you know, the important kind-of … like the CANMAT guidelines, like I can use like certain things when I am explaining to patients. And then they can go and do more reading, right. So, I think it explains everything to them.”

Other practitioners did not feel that the toolkit specifically helped to improve their confidence. These practitioners also felt confident in discussing exercise prior to receiving the toolkit.

### Intervention coherence

In general, the participants demonstrated at some point in the interview that they understood the purpose of the toolkit. Only one participant did not seem to understand its purpose until the post-interview period. She explained a lack of understanding with regards to the intended population to use the toolkit with someone (with mild-moderate depression), and in making a connection between exercise and depression:

“So I don’t know if my population is what would normally use this toolkit … because, my population was not as severe as someone that - and my population has some awareness of the relationship between exercise and depression. But I didn’t actually bring that together with the connection using the toolkit”.

Throughout the post-interview she recognized in hindsight how she could better implement the toolkit in her practice and acknowledged she would do so moving forward through promoting the connection between mood and physical activity and not just recommending exercise.

#### Adoption, modification and dissemination

All study participants expressed that they would continue to use the toolkit and that they would recommend it to colleagues. Participants were asked if they felt that the toolkit needed any additional training to help HCPs use it in their practice (e.g. a webinar, or in-person educational session). Only one participant felt that necessary: “… if I went to a webinar for two hours on just a little bit more of exercise, I’d probably be more confident”. Four participants suggested modifications to the toolkit, whereas two felt that they liked it as is. Modifications included: adding references to the hosting website, adding a mood and activity diary with a monthly calendar, adding a schedule with a monthly calendar, and adding a weekly schedule example with more realistic activities for individuals with severe depression such as getting out of bed or leaving the house.

With regards to dissemination of the toolkit, several strategies to reach practicing HCPs in Canada were provided, including: word of mouth, contacting public health units/ mental health teams/health authorities in British Columbia, faxing primary care offices, attending and presenting at conferences, contacting educational training programs of HCPs, directly mailing to HCPs’ addresses listed on registry bodies, contacting professional registry bodies and reaching out to other specific organizations associated with HCPs in each province.

## Discussion

The primary purpose of this study was to evaluate the acceptability and perceived effectiveness of the ‘Exercise and Depression Toolkit’ in practice. Adopting a hybrid of the Diffusion of Innovation Theory [8] and the Theoretical Framework of Acceptability [13] was useful in determining the factors that may influence adoption. In summary, all HCPs used the toolkit, and for the most part found their interactions when using it with people with depression to be successful. The toolkit was well-liked, and participants viewed it as having relative advantage and perceived effectiveness, compatibility and ethicality, low complexity and trialability (adaptable to use and suit needs). Some participants did not always see the toolkit as having observability when using it, as they could not always detect changes in the individuals they were working with nor did they have an opportunity to observe other practitioners implement the toolkit. Participants identified that the toolkit does have some burden, requiring time and effort to use. Feelings were mixed about the toolkit changing self-efficacy to discuss and recommend exercise – and this was one of the original objectives of the toolkit.

It is promising that the toolkit was well-liked and perceived as having relative advantage and perceived effectiveness. All participants viewed the toolkit as helping them to discuss exercise, and better than previous approaches in doing so. This has been deemed particularly important for the adoption of HCPs’ use of educational resources such as a ‘toolkit’ [12]. This includes the importance of adopters viewing the resource as having an advantage over other resources, and that the evidence-base for the resource is clear [12]. Further, HCPs did view the toolkit as being compatible with their current practice and behaviours. Compatibility may be the most important attribute for provider uptake of an innovation [11].

In this study, participants did not believe using the toolkit took their time away from other priorities (opportunity costs). However, the participants identified that using the toolkit did require time and effort which could negate the feelings of opportunity costs. Pharmacists delivering a mental health promotion program identified concerns about diverting time and money away from other tasks which likely impacted uptake [17]. Lack of time to discuss and recommend exercise has been identified as a barrier by mostly mental health nurses [24–27], likely for similar reasons. While some participants did view the toolkit as being of some burden, they felt that it was worth the extra effort which is promising for future uptake. Further, participants had strong views on the simplicity of the toolkit which may have moderated perceptions of the effort and time to implement the toolkit practice.

There was inconsistency from HCPs in this study about observability. Due to the design of the study, participants were not able to observe actions of their peers using the toolkit which could influence adoption. Thus, observability was considered changes as noted by HCPs in the individuals they were working with when using the toolkit. The time frame of this study was 4 weeks, and the Family Physician in primary care explained this was too short for her to see her patients for follow-up. On the other hand, the Psychotherapist in an outpatient setting was seeing people daily or weekly and was able to observe various changes such as increased activity levels and improved mood. It is also important to consider such differences among participants (e.g. health care provider designation, practice context) when interpreting results. Canadian treatment guidelines recommend the length of an exercise program to see depression treatment effects be 9 weeks and supervised for adherence [1]. Thus, it is likely that it may take more time than 4 weeks to see mood effects and changes, and many individuals with depression in the current study were not able to be referred to a structured supervised program.

The secondary purpose of the study was to attain feedback on the toolkit to determine necessary modifications and help inform national dissemination and uptake strategies. Based on the results of this study, there are no current plans to create additional training for the toolkit. However, recommendations on use of the toolkit and its development are currently being embedded into a training module for exercise professionals to deliver programming to individuals with mental illness inclusive of depression. The toolkit was designed to be something simple and easy to use that would not require further extensive training, and participants in this study all identified this except for one. With regards to dissemination, all recommendations were considered given available funding and time. Various strategies were adopted from the recommendations including: word of mouth (emailing personal HCP contacts and asking them to share with their respective networks), contacting public health units and mental health teams in British Columbia, attending and presenting at conferences, and sharing with professional registry bodies and organizations associated with HCPs. The concerns of burden from participants were also considered in the dissemination. Several psychiatrists, psychologists and some family physicians declined to participate in this study given ‘lack of time’. These health professionals may not be the best target of dissemination efforts in the short-term. Thus, more efforts were put towards targeting and disseminating to allied health professionals and front-line workers such as social workers and occupational therapists.

### Strengths and limitations

A strength of this study is the novel theoretical underpinnings using the DoI theory and the TFA. Using the hybrid approach was helpful for understanding perceptions of important aspects of the toolkit, as well as cognitive and emotional responses to using it. Important factors may have been missed if both theories were not utilized. In terms of limitations, due to the case study design, observability could not be explored in the context of peer behaviour with regards to use of the intervention. Some participants did not always observe changes in people with depression they were working with. The study length was 4 weeks and one participant noted this was not enough time for her to follow-up with patients. Thus, little is known about secondary outcomes of the toolkit for individuals with depression such as mood and physical activity changes. Participants were recruited from a previous study to inform development of the toolkit. While this was done purposely considering the DoI theory, the two participants involved in both studies may have been predisposed to perceive the toolkit favorably. This study had a small and homogeneous sample with regards to gender, ethnicity, and age and participants only represented two provinces across Canada. Participants were physically active and likely receptive to exercise as a treatment consideration. This may explain little impact of the toolkit in enhancing self-efficacy to discuss exercise with individuals. The authors do not know how the toolkit would be received by health care providers who do not have time or interest in considering exercise as a treatment for depression. While there are no current plans to further evaluate the toolkit itself, a team is monitoring perceptions of its implementation within a campus-based exercise intervention for students seeking help for depression.

## Conclusions

Use of the Theoretical Framework of Acceptability and the Diffusion of Innovation Theory was helpful in exploring the use and acceptability of the ‘Exercise and Depression Toolkit’ in practice by health care providers. Overall, the toolkit was found to be acceptable and as having positive innovation attributes: relative advantage and perceived effectiveness, compatibility and ethicality, low complexity and trialability. Future work could address observability and the ability for health care providers to see other providers using it, as well as effectiveness considering outcomes for people with depression such as mood and physical activity changes. The results of this evaluation are promising for uptake and future adoption of the toolkit by health care providers working with adults with depression in Canada.

## Supplementary Information

**Additional file 1.**

## Data Availability

The datasets generated and/or analyzed during the current study are summarized in this manuscript. The raw data is not publicly available due to the possibility of individual privacy being compromised.

## Abbreviations

HCP: Health care provider
DoI: Diffusion of Innovation
TFA: Theoretical Framework of Acceptability
