# Supplementary material for: Evaluation of health care providers’ use of the ‘Exercise and Depression Toolkit’: a case study
Source: BMC Psychiatry. 2021 May 8;21:243. doi: 10.1186/s12888-021-03248-5 (PMC8105951; doi:10.1186/s12888-021-03248-5)
Supplement: Supplementary file 1 — Additional file 1. [file 12888_2021_3248_MOESM1_ESM.docx]

**Interview Guide 1: The Exercise and Depression Toolkit Evaluation Pre-intervention**

My name is Krista, I’m a research assistant and student for the University of British Columbia. Thank you for talking with me today. I have emailed you a consent form explaining your participation in this study. Have you reviewed it?

If No: ask them to read it now, re-send it necessary.

Yes: Do you agree to participate? If participant says no, no interview will be conducted.

I have also emailed you the ‘Exercise and Depression Toolkit’ and the Weekly Log. Have you received these?

If No: Resend it

Yes: You will need both for part of this interview. You can either pull them up on a screen to see, or print it out. Before we continue, do you have these documents available to view? [give time if they do not have it ready].

What we talk about today is kept confidential, and your answers will be stored separate from your name.

We are here to talk about a resource developed for professionals to explore exercise as a treatment option for depression – the ‘Exercise and Depression Toolkit’.

1. In your current clinical practice, do you consider exercise as a treatment for depression? How do you discuss or recommend exercise/physical activity to your clients?
   1. *Prompts: How? How often? What do you discuss? Are they certain clients you discuss it with and not others?*
   2. *Prompt: General physical activity promotion/recommend exercise as a treatment/facilitate any programming? What are you doing currently?*
2. What type of treatment/intervention do you usually provide to adults with depression?
3. What are some of the barriers you face to recommending or discussing exercise as a treatment for depression? What are some of the facilitators you experience to recommending or discussing exercise as a treatment for depression?
4. What are some of the barriers you think may face in using the toolkit in your practice? What will help you to use the toolkit?

TFA: Ethicality-The extent to which the intervention has good fit with an individual’s value system [DoI: Compatibility]

1. Does recommending exercise align with your role as a health care professional (e.g. as a physician, as an occupational therapist etc.)? Why or Why not?
   1. *Prompt: Do you feel this is part of your role?*
2. Do you feel that recommending exercise aligns with your personal beliefs and values? Why or Why not?

TFA: Intervention Coherence-The extent to which the participant understands the intervention and how it works

6. From your perspective, what do you think the Exercise and Depression toolkit is trying to achieve?

TFA: Self-efficacy- the participant’s confidence that they can perform the behaviour required to participate in the intervention

7. How confident do you feel in discussing and recommending exercise as a treatment option for depression?

8. How confident do you feel in using the toolkit to help such discussions?

Before we review the toolkit and the weekly log, is there anything else you would like to add that we did not discuss or that you think is important?

*Provide explanation of the toolkit and its use and answer any questions about its use. * See ‘Intro’ Document and read it to them as they pull up each page of the toolkit.*

Now I will briefly review the weekly log with you and how to fill it out.

*Read instructions on the weekly log.* Do you have any questions as to how to fill this out? Try to fill it out every day, to help you remember any details. Please email it to me at the end of your work week, and if you would like save a blank copy. I will email you with a reminder every Friday to collect your log, and ask if you need me to send you a new blank one. If you would prefer me to call you please let me know.

Thank you so much for your participation thus far.

**Interview guide post-intervention**

Interview Guide 2: The Exercise and Depression Toolkit Evaluation

Hello again just as a reminder, my name is Krista, I’m a research assistant and student for the University of British Columbia. Thank you for talking with me again today.

I have received your weekly logs from this past month. [If not ask participant to send the logs].

Thank you.

What we talk about today is kept confidential, and your answers will be stored separate from your name.

We are here to talk about your experience with using the ‘Exercise and Depression Toolkit’.

1. Please tell me about your experience in general of using the Exercise and Depression toolkit in your practice.
2. When/Why did you use the toolkit?

*Prompt:* What factored into your decision to use the toolkit? (e.g. only certain types of clients- age, gender, severity of depression etc.) *Refer to provider’s weekly log to discuss instances of using the toolkit.

**Theoretical Framework of Acceptability Constructs guided questions**

General acceptability

1. How acceptable do you think the toolkit is for use in your practice?

Affective attitude- how an individual feels about the intervention

1. In general, how do you feel about the ‘Exercise and Depression Toolkit’?
2. What did you like about the toolkit? What did you dislike about the toolkit?
3. Are there any modifications/changes you would make to the toolkit? (e.g. wording, or important content is missing)

Burden- The perceived amount of effort that is required to participate in the intervention

1. How much effort does it take you to use the toolkit? *OR* Do you feel the toolkit requires a lot of time and effort to use in practice?

Opportunity Costs- The extent to which benefits, profits or values must be given up to engage in the intervention

1. Do you think using the toolkit in practice has interfered with your other priorities as a health care provider?

*Prompt:* e.g. takes time away from other discussions, other treatments

Perceived effectiveness- the extent to which the intervention is perceived to be likely to achieve its purpose [DoI: Relative Advantage]

1. How has the toolkit helped you (if at all) to engage in discussions with adults with depression about exercise/ exercise as a treatment option?

*Prompt:* Use their log to discuss perceived success

Ethicality-The extent to which the intervention has good fit with an individual’s value system [DoI: Compatibility]

1. Does recommending exercise align with your role as a health care professional (e.g. as a physician, as an occupational therapist etc.)? Why or Why not?
2. Does recommending exercise aligns with your personal beliefs and values? Why or Why not?

*a. Prompt:* Has this changed from before you used the toolkit in practice?

Intervention Coherence-The extent to which the participant understands the intervention and how it works

1. From your perspective, what do you think the toolkit is trying to achieve?

*Prompt:* Has this changed from before you used the toolkit in practice?

Self-efficacy- the participant’s confidence that they can perform the behaviour required to participate in the intervention

13. How confident do you feel to continue to use the toolkit to discuss and recommend exercise as a treatment for depression? **prompt: compare to pre interview*

14. How has the toolkit has helped improve your confidence to discuss and recommend exercise as a treatment option (if at all)? **prompt: compare to pre interview*

**Other Questions:**

1. What was the most common end point when you used the toolkit?

*Prompt:* (page 4. ‘Moving More’: Referral to an exercise program, Engage in PA counselling with yourself or another HCP, Discuss exercise at a later date)?

*Prompt (check logs and pre-interview)* For those that did not have access or refer to a structured supervised exercise program: Please explain

**Diffusion of Innovation Theory**

Simplicity:

1. Do you feel the toolkit is easy to understand and use?

Observability:

1. Are you able to observe any positive changes in your clients from using the toolkit? *Prompt:* E.g. changes in mood, increased physical activity levels, engagement in an exercise program

Trialability:

1. Do you feel you could adapt the toolkit to suit your needs? *Prompt*: e.g. only use certain pages like giving the Guidelines at a glance, or supplementary handouts etc.

**Adoption-** intention, initial decision or action to attempt or utilize an innovation or EBP

1. Will you continue to use the toolkit to recommend and discuss exercise as a treatment for depression in your practice? *Prompt*: Why or Why not?
2. What do you think you will primarily use the toolkit for?
   1. *Prompt: Discussing exercise as a treatment, PA promotion in general*
3. Would you recommend it to other clinicians/colleagues? *Prompt:* Why or Why not?

**Dissemination**

1. We hope to share the toolkit with health care providers across Canada to use. Do you have any suggestions for this dissemination of the toolkit?
2. What do you feel the toolkit needs in addition to support its use by other health care providers?
   1. *Prompts: Educational workshops for HC (in-person, online webinar), Conferences, Media campaigns/ Social media or marketing campaigns, Promotional video, Key champions/opinion leaders to connect with*

1. What are some recommended or trusted channels/networks to share the toolkit through?

*Prompt: Who would you want to hear about the toolkit from? (E.g. individual such as manager or co-worker, professional licensing body, agencies, etc.)*

This brings us to the end of the interview. Before we finish, is there anything else you would like to add that you feel is important or that we have not talked about?

You will be sent an email with a gift card for your participation shortly. If you do not receive it please follow-up with me. We will contact you when the toolkit is available for public use unless you do not wish to be contacted.

Thank you so much for participation in this study.

**Weekly provider log**

**Weekly Provider Log**

ID:

Date today:

- Please **check** what day of the week you used the toolkit (Please indicate the number of times you used it if you used it more than once in one day, and if you used it with more than one client), and if you referred any clients to an exercise program.

*Please rate your Perceived Success of your interaction with your client on a scale of 1-7 (1 being extremely low success, and 7 being extremely high success). *Consider usefulness, client receptiveness, and satisfaction. Please also provide the number of clients (adults) with depression you saw that day.*

*Please provide any additional comments you have about your experience using the toolkit (e.g. likes, dislikes, etc.).*

*If you did not use the toolkit, please indicate a reason(s) in the comment section.*

|  | MON | TUES | WEDS | THURS | FRI | SAT | SUN |
| --- | --- | --- | --- | --- | --- | --- | --- |
| Used the full toolkit |  |  |  |  |  |  |  |
| Used part of the toolkit (only collaborative pages 1-4 or additional supplementary handouts)  **please specify* |  |  |  |  |  |  |  |
| Number of individuals with depression seen |  |  |  |  |  |  |  |
| Perceived Success (1-7) |  |  |  |  |  |  |  |
| Referral to structured and supervised exercise program |  |  |  |  |  |  |  |

If you used the toolkit and did not refer to an exercise program, please explain what you did instead:

**Additional Comments** *(likes, dislikes, usefulness, satisfaction, details of exercise program etc.):*
